# CREB1-induced miR-1204 promoted malignant phenotype of glioblastoma through targeting NR3C2

**DOI:** 10.1186/s12935-020-01176-0

**Published:** 2020-04-07

**Authors:** Xinli Zhao, Fazheng Shen, Jiwei Ma, Shupeng Zhao, Lei Meng, Xiangyang Wang, Shufeng Liang, Jianing Liang, Chaoshuai Hu, Xinzhong Zhang

**Affiliations:** Department of Neurosurgery, The First Affiliated Hospital of Xinxiang Medical University, 88 Health Road, Weihui, 453100 Henan China

**Keywords:** miR-1204, Glioblastoma, CREB1, NR3C2

## Abstract

**Background:**

Glioblastoma (GBM) is a subclass of brain malignancy with unsatisfactory prognosis. MicroRNAs (miRNAs) are a group of non-coding RNAs (ncRNAs) that exert key function on tumorigenesis and tumor development.

**Purposes:**

The purpose of this work was to unravel the biological behavior and mechanism of miR-1204 in GBM.

**Methods:**

Expressions of miR-1204, NR3C2 and CREB1 were detected by RT-qPCR and western blot. Proliferation and apoptosis of GBM cells were detected by CCK-8, colony formation, caspase-3 activity and TUNEL assays. Molecular interplays were examined by ChIP, RIP, and luciferase reporter assays.

**Results:**

MiR-1204 level was elevated in GBM cell lines. Functionally, miR-1204 aggravated cell proliferation whereas suppressed cell apoptosis in GBM cells. Mechanistically, cAMP Responsive Element Binding Protein 1 (CREB1) bound to the promoter of miR-1204 and activated the transcription of miR-1204. Furthermore, miR-1204 targeted and inhibited Nuclear receptor subfamily 3 group C member 2 (NR3C2), a tumor suppressor gene in GBM cells. Rescue assays indicated that NR3C2 participated in the regulation of miR-1204 on the malignant phenotype of GBM cells.

**Conclusions:**

We observed for the first time that CREB1-induced miR-1204 promoted malignant phenotype of GBM through targeting NR3C2, indicating that miR-1204 acted as a novel oncogenic miRNA in GBM.

## Background

Glioblastoma (GBM) is identified as the most prevalent primary malignant brain tumor with high risk of invasion in adults [1]. Although the advancements have been achieved in clinical standard therapies, such as surgery, radiation, and chemotherapy over the past few decades, the median survival of GBM remains shorter than 2 years [2]. It is believed that tumor heterogeneity is at least part of the reason for poor prognosis in GBM, which was firstly proved by histopathological findings [3]. More importantly, GBM has a high degree of polymorphism in cytology. On one hand, the constituent cells of GBM exhibit high variability in size and shape, and on the other hand, they possess large bizarre nuclei or multinucleated characteristics [4].

The heterogeneity of GBM has now been demonstrated in many aspects. And we have increasingly recognized that intratumoral genetic heterogeneity is the core of GBM biology, which may pose a huge challenge to the improvement of therapeutically effective methods [5]. Initially, heterogeneity within the tumor is confirmed by the analysis of tumor volume, revealing changes in regional copy number, isomer cell mutation or gene expression difference [6, 7]. Thereafter, heterogeneity of GBM is further supported by the findings that spatially differently distributed fragments extracted from the same tumor are corresponded to varying GBM molecular subtypes [7]. The abovementioned findings are considered as the key step to understand intratumoral heterogeneity. Considering that each cell contained in a single tumor may have a unique gene expression feature under different conditions, they are worthy of more careful examination at higher resolution. Therefore, further investigation on the molecular regulatory mechanism of GBM progression is necessary to improve the prognosis of GBM patients.

As a cluster of non-coding RNAs (ncRNAs) with the length of around 22 nucleotides, microRNAs (miRNAs) have been proved to have prognostic and therapeutic significance in diverse cancers [8–11]. And multiple researchers have been dedicated to unraveling the crucial function of dysregulated miRNAs on cancer progression and identifying novel miRNAs as potential biomarkers for cancer diagnosis and treatment [12–14]. MiRNAs function through interacting with messenger RNAs (mRNAs) at the 3′ untranslated region (3′UTR) to restrain gene expression [15]. They can serve as oncogenes or tumor-suppressive genes in cancers by targeting genes with different functions [16–19]. Over the past decades, the regulation of miRNAs on GBM progression has been accumulatively studied and reported. For example, miR-1179 suppresses GBM proliferation and cell cycle through targeting E2F5 [20]. MiR-133a promotes TRAIL resistance in glioblastoma through repressing DR5 and NF-κB pathway [21]. MiR-137 suppresses CXCL12 expression, inhibiting GBM progression [22]. In addition, it has been reported that miRNAs are related to recurrence-free survival in GBM patients [23]. Further, the oncogenic role of miR-1204 in some cancers has been validated. For instance, miR-1204 contributes to cell growth in ovarian squamous cell carcinoma via increasing glucose uptake [24]. MiR-1204 promotes epithelial–mesenchymal transition (EMT) and metastasis in breast cancer by targeting VDR [25]. MiR-1204 facilitates the development of hepatocellular carcinoma by stimulating MAPK and c-JUN/AP1 pathway via targeting ZNF418 [26]. MiR-1204 induces the proliferation of non-small-cell lung cancer cells through modulating PITX1 expression [27]. However, the specific role of miR-1204 in GBM remains unclear, thus arousing our interest in exploring whether and how miR-1204 regulates the biological process in GBM cells, including cell proliferation and cell apoptosis.

Recent studies have shown that miRNAs can be transcriptionally activated by certain transcription factors (TFs). For example, miR-140-5p is transcriptionally activated by vitamin D receptor (VDR) in bone development [28]. The proto-oncogenic miR-744 is induced by c-JUN at transcriptional level in non-small cell lung cancer [29]. Additionally, cAMP responsive element binding protein 1 (CREB1), known as a transcription factor participating in the metabolism and DNA repair [30], has been demonstrated to be cancer-promoting in various malignancies, including GBM [31, 32]. Further, existing studies of cancers have reported that CREB1 could induce the transactivation of oncogenic miRNAs, such as miR-23a and miR-302a [30, 33]. Nevertheless, the relation between CREB1 and miR-1204 in GBM has not been uncovered.

Nuclear receptor subfamily 3 group C member 2 (NR3C2) is defined as a mineralocorticoid receptor gene which translates the mineralocorticoid receptor (MR), a transcription factor responsible for electrolyte balance [34]. Previous reports have shown that MR downregulation predicts poor prognosis in lung cancer and colon cancer [35, 36]. Later, some studies have revealed the tumor-suppressive role of NR3C2 in cancer progression, such as in pancreatic cancer and clear cell renal cell carcinoma [37, 38]. However, the underlying role of NR3C2 in GBM has never been investigated.

The purpose of the present study was to investigate the function and regulation mechanism of miR-1204 in GBM.

## Methods

### Cell culture

Four glioma cell lines including A172, LN229, U251 and U87, and a normal human astrocyte cell line (NHA) were obtained from the Institute of Biochemistry and Cell Biology of the Chinese Academy of Sciences (Shanghai, China). And 293T cell line were procured from American Type Culture Collection (ATCC, Manassas, VA, USA). All these cells were maintained in DMEM medium (Thermo Fisher Scientific, Inc., Waltham, MA, USA) at 37 °C with 5% CO_2_. The DMEM culture medium contained 10% fetal bovine serum (FBS; Thermo Fisher Scientific, Inc., Waltham, MA, USA), and 1% penicillin (100 U/ml)/streptomycin (100 U/ml).

### RNA isolation and real-time quantitative polymerase chain reaction (RT-qPCR)

Total RNA was extracted from cultured A172 and U251 cells by TRIzol reagent (Invitrogen, Carlsbad, CA, USA) as per the standard method provided by supplier. Next, complementary DNAs (cDNAs) were synthesized with a Reverse Transcription Kit (Invitrogen). By use of the FastStart Universal SYBR Green Master (Vazyme Biotech), quantitative real-time PCR (RT-qPCR) was carried out by an ABI 7500 Fast Real-Time PCR system in light of the specific protocol. RT-qPCR measured the expression level of genes, with GAPDH or U6 as the endogenous control. Comparative quantification was examined by 2^−ΔΔCT^ method with at least three independent experiments. The related PCR primers were presented in Table 1.

**Table 1:** Related PCR primers and primers for plasmids

Gene name	PCR primers (5′-3′)
miR-1204	F:GUGGCCUGGUCUCCAUUACC R:CTCTACAGCTATATTGCCAGCCAC
CREB1	F:TGCAACATCATCTGCTCCCA R:CTGAATAACTGATGGCTGGGC
NR3C2	F:GAGAGCCCACATTGCTAGCA R:GCCCTGCTGGAATCAACTCT
GAPDH	F:GAGAAGGCTGGGGCTCATTT R:AGTGATGGCATGGACTGTGG
U6	F:CTCGCTTCGGCAGCACA R:AACGCTTCACGAATTTGCGT

Plasmid name	Related sequences (5′-3′)
NC mimic	UCCCGGGGUGGUCUCCAUUAU
miR-1204 mimic	UCGUGGCCUGGUCUCCAUUAU
NC inhibitor	AUAAUGGAGACCACCCCGGGA
miR-1204 inhibitor	AUAAUGGAGACCAGGCCACGA
sh-NC	CCGCCAGAAATGCAAATTCTTATTCCTCGAGGAATAAGAATTTGCATTTCTGTTTTTG
sh-CREB1#1	CCGCCAACTATTGCAGAAAGTGAAGCTCGAGCTTCACTTTCTGCAATAGTTGTTTTTG
sh-CREB1#2	CCGCGAAAATTTTGAATGACTTATCCTCGAGGATAAGTCATTCAAAATTTTCTTTTTG
sh¬NC	CCGCCAGAAACAACAAAAAGTAAAACTCGAGTTTTACTTTTTGTTGTTTCTGTTTTTG
sh¬NR3C2#1	CCGCGAATAATAGTCTTTATCATCCCTCGAGGGATGATAAAGACTATTATTCTTTTTG
sh¬NR3C2#2	CCGCGAAACACAGCTTACGTTGACACTCGAGTGTCAACGTAAGCTGTGTTTCTTTTTG
pcDNA3.1/CREB1	ATGACCATGGAATCTGGAGCCGAGAACCAGCAGAGTGGAGATGCAGCTGTAACAGAAGCTGAAAACCAAC AAATGACAGTTCAAGCCCAGCCACAGATTGCCACATTAGCCCAGGTATCTATGCCAGCAGCTCATGCAAC ATCATCTGCTCCCACCGTAACTCTAGTACAGCTGCCCAATGGGCAGACAGTTCAAGTCCATGGAGTCATT CAGGCGGCCCAGCCATCAGTTATTCAGTCTCCACAAGTCCAAACAGTTCAGATTTCAACTATTGCAGAAA GTGAAGATTCACAGGAGTCAGTGGATAGTGTAACTGATTCCCAAAAGCGAAGGGAAATTCTTTCAAGGAG GCCTTCCTACAGGAAAATTTTGAATGACTTATCTTCTGATGCACCAGGAGTGCCAAGGATTGAAGAAGAG AAGTCTGAAGAGGAGACTTCAGCACCTGCCATCACCACTGTAACGGTGCCAACTCCAATTTACCAAACTA GCAGTGGACAGTATATTGCCATTACCCAGGGAGGAGCAATACAGCTGGCTAACAATGGTACCGATGGGGT ACAGGGCCTGCAAACATTAACCATGACCAATGCAGCAGCCACTCAGCCGGGTACTACCATTCTACAGTAT GCACAGACCACTGATGGACAGCAGATCTTAGTGCCCAGCAACCAAGTTGTTGTTCAAGCTGCCTCTGGAG ACGTACAAACATACCAGATTCGCACAGCACCCACTAGCACTATTGCCCCTGGAGTTGTTATGGCATCCTC CCCAGCACTTCCTACACAGCCTGCTGAAGAAGCAGCACGAAAGAGAGAGGTCCGTCTAATGAAGAACAGG GAAGCAGCTCGAGAGTGTCGTAGAAAGAAGAAAGAATATGTGAAATGTTTAGAAAACAGAGTGGCAGTGC TTGAAAATCAAAACAAGACATTGATTGAGGAGCTAAAAGCACTTAAGGACCTTTACTGCCACAAATCAGA T

### Cell transfection

Ahead of transfection, A172 and U251 cells were plated in six-well plates and incubated for 24 h. Thereafter, transfections were performed using Lipofectamine 2000 (Invitrogen). MiR-1204 mimic, miR-1204 inhibitor and matched negative controls (NC mimic, NC inhibitor) were synthesized by Shanghai GenePharma Co. (China). CREB1 was overexpressed using the pcDNA3.1 vector and the pcDNA3.1 empty vector was used as the control, and the vectors were constructed by ZonHon Biopharma Institute, Inc (Changzhou, Jiangsu, China). Short hairpin RNAs (shRNAs) targeting CREB1 (sh-CREB1#1, sh-CREB1#2) or NR3C2 (sh-NR3C2) and negative control shRNA (sh-NC) were obtained from GenePharma Corporation (Suzhou, Jiangsu, China). Lastly, the efficiency of transfection was examined by RT-qPCR 48 h after transfection. Related primers for plasmids were presented in Table 1.

### Cell Counting Kit-8 (CCK-8) assay

A172 and U251 cells were seeded in 96-well plates and then cultured for 0, 24, 48, 72, 96 h. Later, each well was added with 10 μl CCK-8 reagent. Cell viability of glioma cells was determined by CCK-8 assay kit (Dojindo, Kumamoto, Japan). After 4 h of incubation, the absorbance of 450 nm was monitored in the dark by a microplate reader (EL340; Bio-Tek Instruments, Hopkinton, MA, USA).

### Colony formation assay

A172 or U251 cells with indicated transfections were evenly placed into a fresh six-well plate (500 cells per well), and then maintained in DMEM culture medium supplemented with 10% FBS. Meanwhile, the medium was changed every 3 days. After 14 days, cells were washed with PBS, and were fixed with 4% formaldehyde for 10 min at room temperature. Next, cells were stained with 0.1% crystal violet for 5 min. Finally, colonies (≥ 50 cells) were photographed and counted through a microscope (TE2000-U; Nikon, Tokyo, Minato City, Japan).

### Western blot assay

Firstly, total proteins were obtained from cells in RIPA buffer (Solarbio Science & Technology Co. Ltd., Beijing, China) containing proteinase inhibitor. Next, a BCA protein assay kit was adopted to quantify protein, SDS-PAGE gels were used to separate protein, and then protein samples were transferred onto PVDF membranes (EMD Millipore, Billerica, MA, USA) with 5% skim milk. Subsequently, the membrane was co-incubated with anti-NR3C2 (1:1000, ab2774) and anti-GAPDH (1:1000, ab8245) at 4 °C all night, and then incubated with secondary antibody rabbit anti-sheep IgG H&L (HRP) (1:2000, ab6747) in a dark room at 37 °C for 1 h. Antibodies used in Western blot assay were acquired commercially from Abcam (Cambridge, USA). Finally, the immunoblots were subjected to enhanced chemiluminescence reagent. GAPDH was used as the loading control.

### Luciferase reporter assay

293T cell line from American Type Culture Collection (ATCC, Manassas, VA, USA) was incubated in 24-well plates with culture medium. For the binding of CREB1 to the miR-1204 promoter, the binding region was subjected to PCR amplification and cloned into the pGL3 vector (Promega, Madison, WI, USA). For the binding of miR-1204 to NR3C2 3′-UTR, cDNA fragment containing the predicted miR-1204 binding sites was amplified by PCR and then inserted to the pGL3 vector. Then the NR3C2 wild-type construct (NR3C2-WT) was generated. The construct with the mutation of miR-1204 binding sites of NR3C2 3′-UTR (NR3C2-MUT) was obtained using Quick Change Site-Directed Mutagenesis Kit (Agilent, Roseville City, CA, USA). Co-transfection of the abovementioned reporter vectors with transfection plasmids into cells was carried out using Lipofectamine 2000 (Invitrogen), respectively. Luciferase activity was tested by a Luciferase Reporter Assay System (Promega, Madison, WI, USA) after 48 h of transfection.

### RNA immunoprecipitation (RIP) assay

Magna RIP RNA-Binding Protein Immunoprecipitation kit (Millipore, Bedford, MA) was used to perform RIP assay. A172 or U251 cells lysed in RIP lysis buffer were treated with anti-AGO2 or anti-IgG and magnetic beads overnight at 4 °C. Then, the immunoprecipitated RNA was isolated, purified using TRIzol regent and detected by RT-qPCR.

### Chromatin immunoprecipitation (ChIP) assay

Using the EZ-Magna ChIP kit (EMD Millipore, Billerica, MA, USA), ChIP assay for miR-1204 promoter analysis was performed. Briefly, A172 and U251 cell lines were cross-linked by use of one percent of formaldehyde solution under the room temperature for 10 min, and then quenched through glycine. DNA fragments were obtained via sonication. The lysate with anti-CREB1 or IgG antibody was immunoprecipitated. Finally, precipitated chromatin DNA was analyzed by RT-qPCR.

### Caspase-3 activity assay

Caspase-3 activity reagent (Beyotime Institute of Biotechnology, China) was used to determine the activity of caspase-3. Firstly, total cell proteins were extracted from cultured A172 and U251 cell lines. After that, proteins were diluted to final volume, and then mixed with caspase-3 substrate for 3 h. Then, hydrolyzed Ac-DEVD-pNA and free pNA released by caspase-3 was detected at 405 nm.

### Terminal-deoxynucleoitidyl transferase mediated nick end labeling (TUNEL) assay

By use of TUNEL Apoptosis Kit (Roche Molecular Biochemicals, Mannheim, Germany), the apoptotic ability of A172 and U251 cells was assessed. The transfected cells were cultured in six-well plates for 48 h, and the residual liquid was washed and re-suspended with PBS. TUNEL reaction mixture added with 70% ethanol was pre-cooled by ice and was applied to cultivate cells for 2 h at 37 °C. Lastly, apoptotic cells were observed by fluorescence microscopy (OLYMPUS IX71; Olympus Corporation, Tokyo, Japan).

### Statistical analysis

In this study, SPSS 17.0 software package (Chicago, Illinois, USA) was used for the statistical analyses. Data were presented as mean ± standard deviation (SD). Student’s t-test or ANOVA was performed for comparisons among groups. Experiments were all repeated at least three times as independent experiment.

## Results

### MiR-1204 was upregulated in GBM, promoted proliferation and hampered apoptosis in GBM

**Fig. 1:**
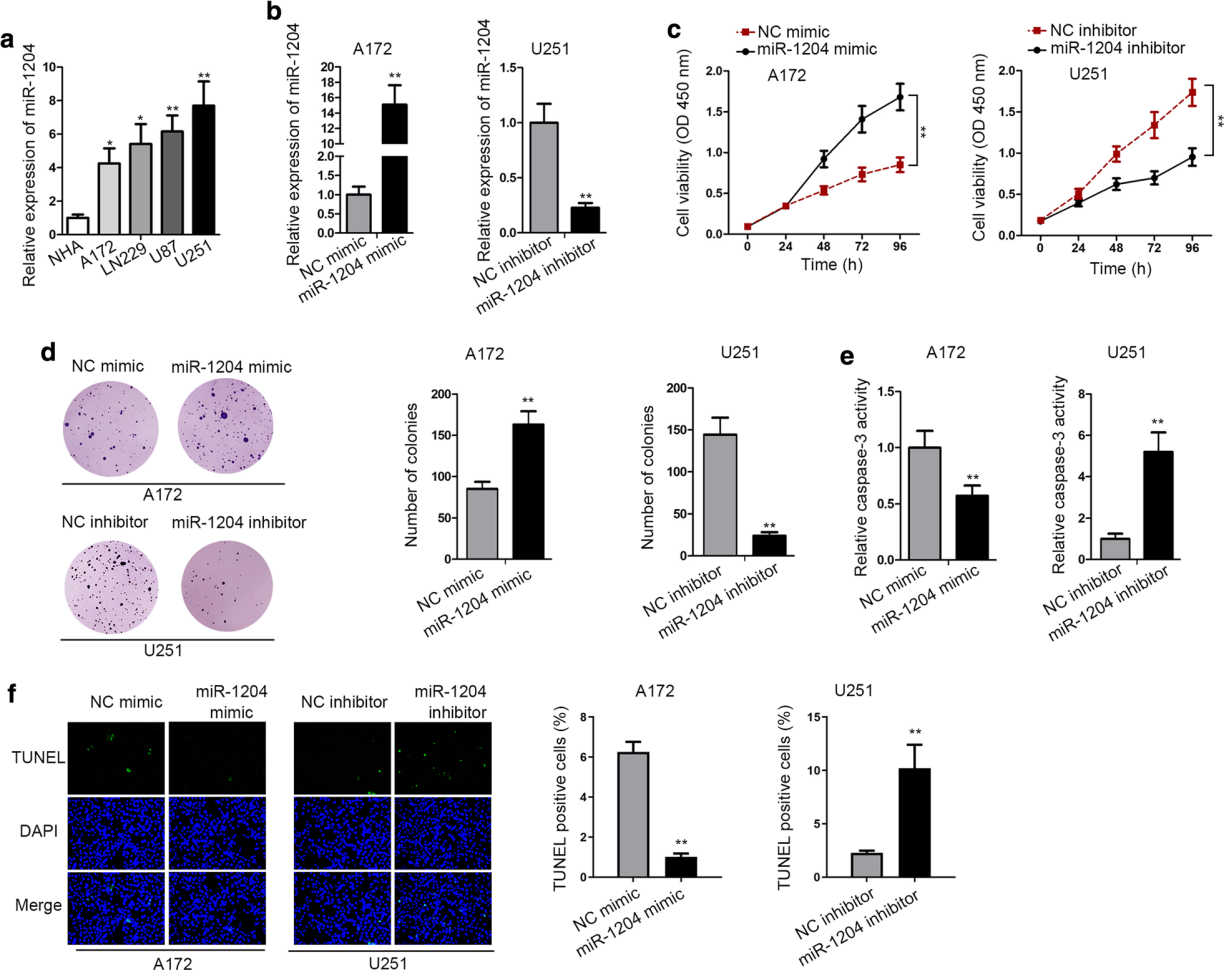
MiR-1204 was highly expressed in GBM and promoted GBM. **a** The upregulation of miR-1204 in GBM cells was confirmed by RT-qPCR. **b** The overexpression efficiency of miR-1204 in A172 cells and the knockdown efficiency of miR-1204 in U251 cells were confirmed by RT-qPCR. **c**, **d** The proliferation of transfected A172 and U251 cells was examined by CCK-8 and colony formation assays. **e**, **f** The apoptosis of transfected cells was analyzed by caspase-3 and TUNEL assays. U6 served as internal control for miR-1204 expression detection. *P < 0.05, **P < 0.01

To explore the potential role of miR-1204 in GBM, we first examined the expression of miR-1204. RT-qPCR data revealed that miR-1204 level was upregulated in GBM cell lines versus normal human astrocyte cell line. And among these GBM cell lines, U251 cells exhibited the highest miR-1204 expression, whereas A172 cells exhibited the lowest miR-1204 expression (Fig. 1a). Next, the biological impact of miR-1204 on GBM cells was investigated through gain-of-function assays in A172 cells and loss-of-function assays in U251 cells. Prior to functional assays, we applied RT-qPCR to determine overexpression efficiency of miR-1204 in A172 cells and the knockdown efficiency of miR-1204 in U251 cells (Fig. 1b). Then, CCK-8 assay demonstrated that overexpression of miR-1204 facilitated the proliferation of A172 cells, while miR-1204 inhibitor exerted the opposite influence on U251 cells (Fig. 1c). Likewise, the number of colonies was increased from 85 to 163 when overexpressing miR-1204 in A172 cells, and the number of colonies was decreased from 144 to 24 under miR-1204 knockdown in U251 cells (Fig. 1d). Subsequently, the apoptosis of A172 and U251 cells was analyzed via caspase-3 activity and TUNEL analyses. Consequently, miR-1204 mimic reduced the caspase-3 activity in A172 cells, whereas miR-1204 inhibitor induced the caspase-3 activity in U251 cells (Fig. 1e). Similarly, TUNEL-positive cells were decreased upon the overexpression of miR-1204 in A172 cells, whereas increased upon the depletion of miR-1204 in U251 cells (Fig. 1f). In conclusion, miR-1204, which was highly expressed in GBM cells, promoted cell proliferation and reduced cell apoptosis.

### MiR-1204 was transcriptionally activated by CREB1 in GBM

**Fig. 2:**
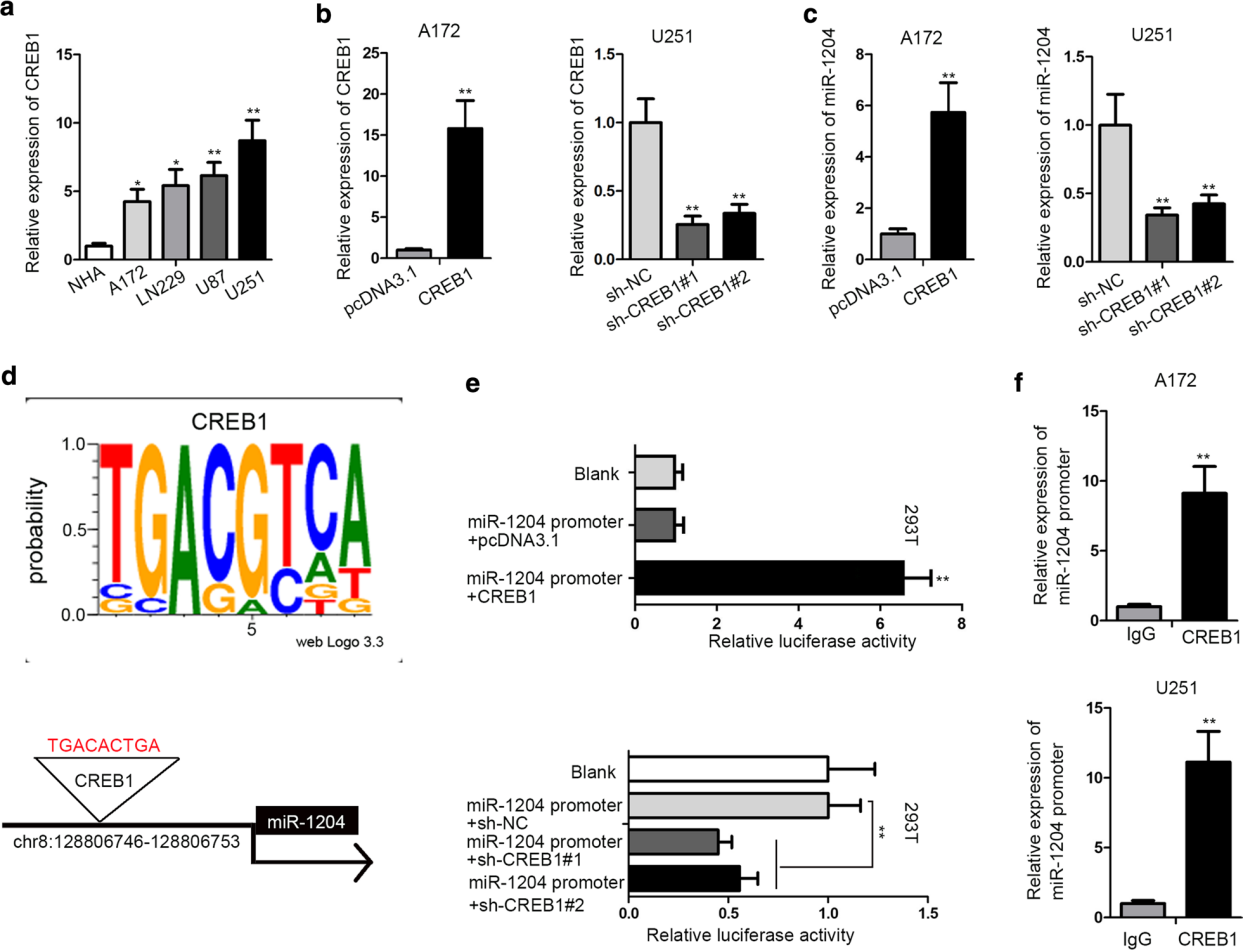
MiR-1204 was transcriptionally activated by CREB1 in GBM. **a** The upregulation of CREB1 in GBM cells was confirmed by RT-qPCR. **b** The overexpression efficiency of CREB1 in A172 cells and the knockdown efficiency of CREB1 in U251 cells were confirmed by RT-qPCR. **c** MiR-1204 expression under the overexpression and knockdown of CREB1 in A172 and U251 cells were detected by RT-qPCR. **d** The DNA motif of CREB1 and the CREB1 binding site on miR-1204 promoter were obtained from miRGen. **e** Luciferase reporter assay was used to detect the impact of CREB1 on miR-1204 transcription. **f** ChIP assay was used to confirm the binding capacity between CREB1 and miR-1204 promoter. GAPDH was used as internal control for CREB1 expression detection, and U6 was used as internal control for miR-1204 expression detection. *P < 0.05, **P < 0.01

Then, we tried to explain the upregulation of miR-1204 in GBM. Recently, the transcriptional regulation of miRNA expression by transcription factor has been delineated by multiple studies [28, 29]. Hence, we aimed to identify the transcription factor targeting miR-1204 promoter. With the aid of miRGen (http://carolina.imis.athena-innovation.gr/diana_tools/web/index.php?r=mirgenv3%2Findex), we found that CREB1 was a potent transcription factor for miR-1204. In addition, it has been manifested by previous studies that CREB1 is a transcriptional factor and exerts carcinogenic role in tumors [31, 32]. Besides, CREB1 can activate certain oncogenic miRNAs through binding to their promoters [30, 33]. Accordingly, we hypothesized that miR-1204 could be transcriptionally regulated by CREB1. Results of RT-qPCR depicted that CREB1 expression was upregulated in GBM cell lines (Fig. 2a). To detect the modulation of CREB1 on miR-1204 expression, we overexpressed CREB1 in A172 cells and knocked it down in U251 cells (Fig. 2b). We observed that CREB1 overexpression elevated miR-1204 level in A172 cells and depletion of CREB1 led to the opposite result (Fig. 2c). Moreover, the DNA motif of CREB1 and the CREB1 binding site on miR-1204 promoter were obtained through miRGen (Fig. 2d). And then luciferase reporter assay showed that overexpression of CREB1 enhanced the luciferase activity of miR-1204 promoter reporter, while downregulation of CREB1 suppressed the luciferase activity of miR-1204 promoter reporter (Fig. 2e). Further, ChIP assay confirmed that miR-1204 was enriched in anti-CREB1 group (Fig. 2f). Altogether, miR-1204 was transcriptionally activated by CREB1 in GBM.

### MiR-1204 targeted NR3C2 to prevent its expression in GBM

**Fig. 3:**
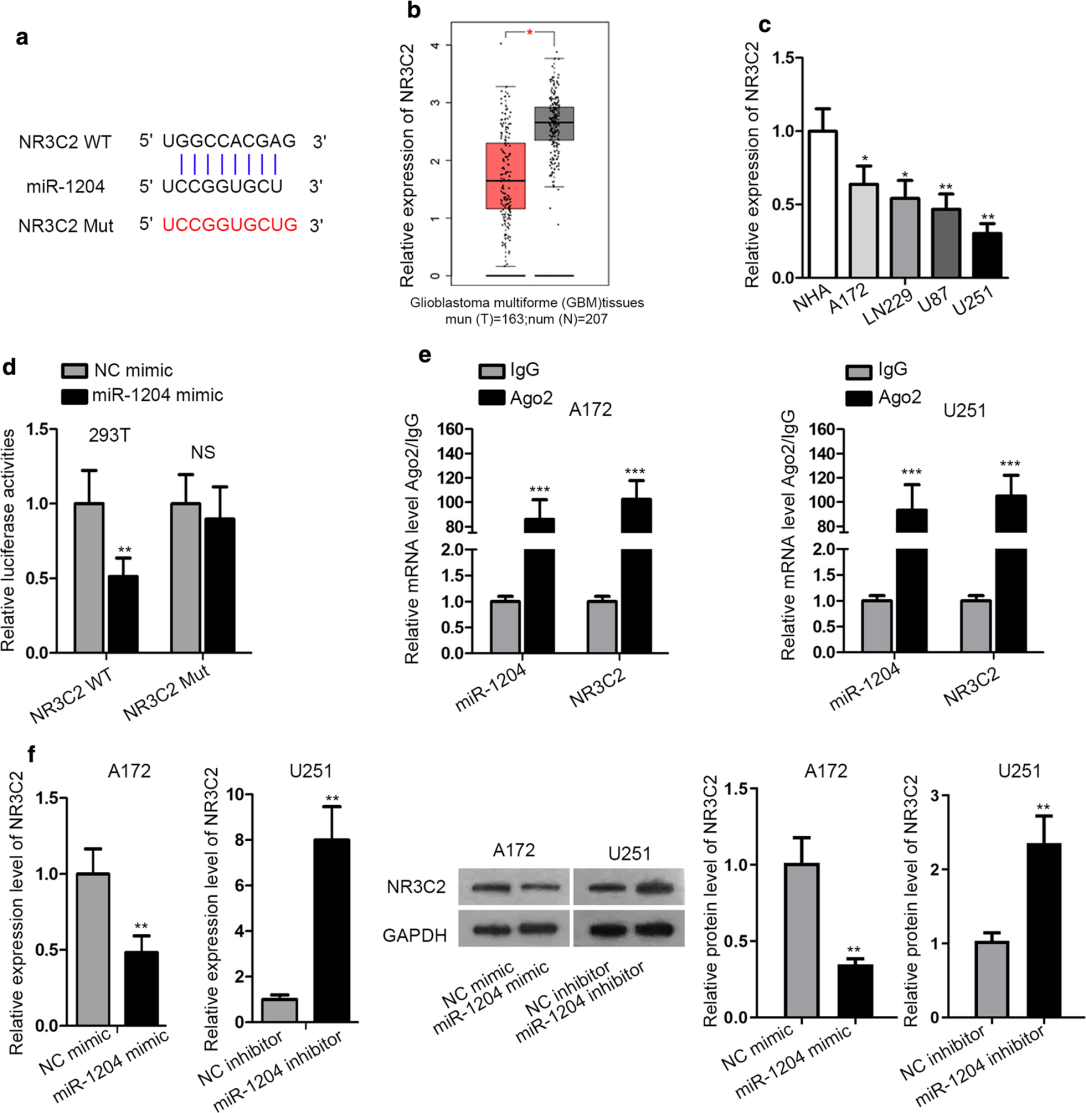
MiR-1204 targeted NR3C2 to repress its expression in GBM. **a** Binding sequences between NR3C2 and miR-1204 were obtained from miRDB and the mutant sequences were designed. **b** Downregulation of NR3C2 in GBM samples was obtained from GEPIA. **c** Downregulation of NR3C2 in GBM cell lines was confirmed by RT-qPCR. **d**, **e** Luciferase reporter assay and RIP assay were used to detect the interaction between miR-1204 and NR3C2 mRNA. *NS* no significance. **f** NR3C2 expression under the overexpression and knockdown of miR-1204 was detected by RT-qPCR and western blot assays. GAPDH was used as internal control for NR3C2 expression detection, and U6 was used as internal control for miR-1204 expression detection.*P < 0.05, **P < 0.01, ***P < 0.001

Subsequently, the downstream mechanism of miR-1204 in GBM was investigated. MiRNAs are largely recognized as the negative modulators of gene expression by interacting with messenger RNAs (mRNAs) at 3′ untranslated regions [15], so we searched miRDB (http://mirdb.org/) for the putative target gene of miR-1204. We identified that NR3C2 was potentially targeted by miR-1204. NR3C2 has been reported to present tumor-repressing behaviors in multiple cancers [37, 38], but it has never been investigated in GBM. Hence, we picked NR3C2 for further exploration. The binding sequences between miR-1204 and NR3C2 were presented in Fig. 3a. Moreover, through GEPIA (http://gepia.cancer-pku.cn/), we discovered that the expression of NR3C2 was significantly downregulated in GBM samples compared with the normal tissues (Fig. 3b). Then RT-qPCR analysis verified the downregulated level of NR3C2 in GBM cell lines (Fig. 3c). Later, luciferase reporter assay manifested that the luciferase activity of NR3C2 WT reporter, rather than NR3C2 Mut reporter, was attenuated by miR-1204 mimic (Fig. 3d). Afterwards, we observed that NR3C2 and miR-1204 were co-immunoprecipitated by AGO2 antibody (Fig. 3e), indicating the binding between miR-1204 and NR3C2 in in A172 and U251 cells. Additionally, overexpression of miR-1204 inhibited NR3C2 mRNA and protein levels in A172 cells, and miR-1204 knockdown elevated NR3C2 expression in U251 cells (Fig. 3f). In a word, miR-1204 targeted NR3C2 to downregulate its expression in GBM.

### MiR-1204 drove GBM cell proliferation by inhibiting NR3C2 expression

**Fig. 4:**
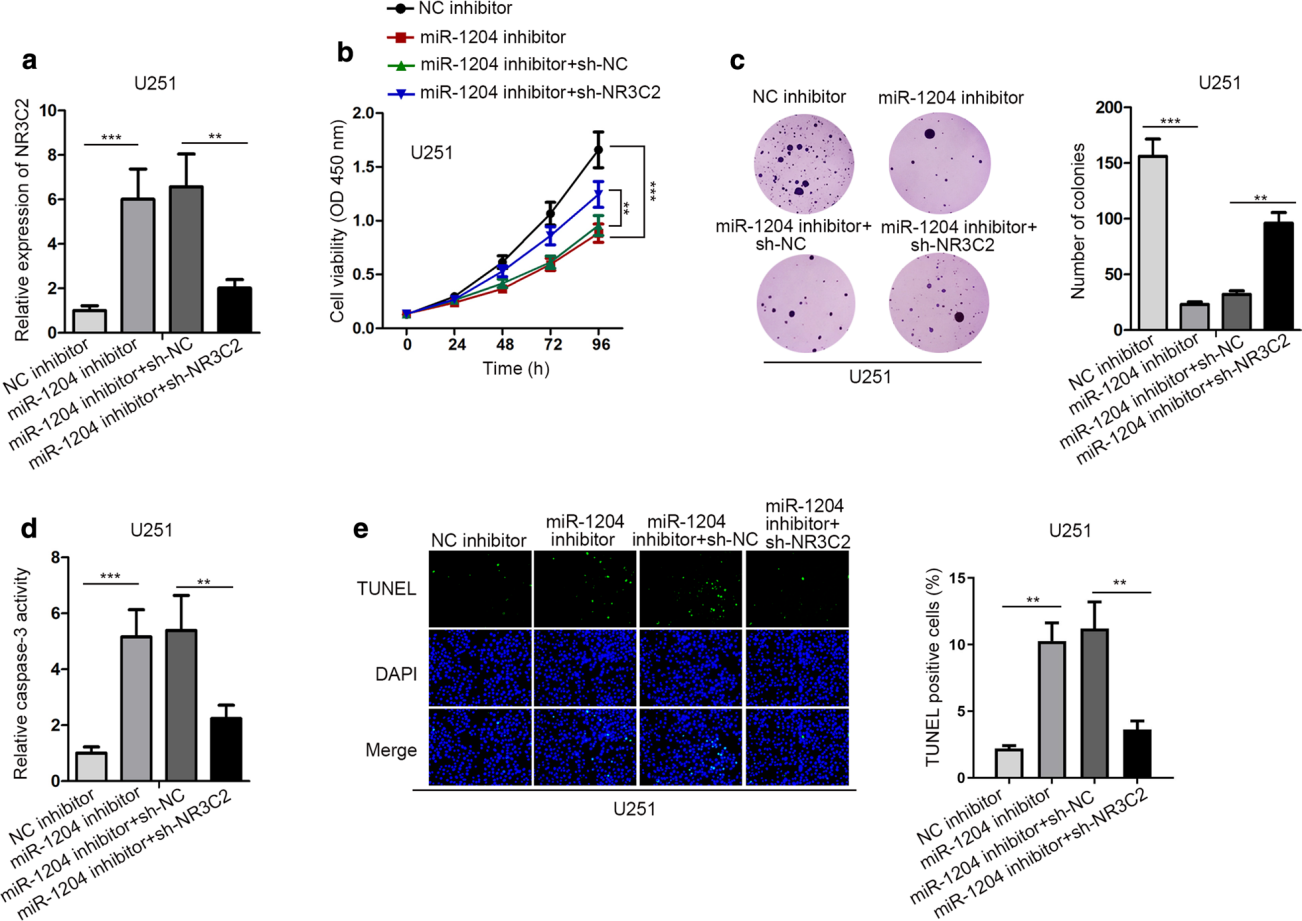
MiR-1204 drove GBM cell proliferation by inhibiting NR3C2 expression. U251 cells were transfected with NC inhibitor, miR-1204 inhibitor, miR-1204 inhibitor + sh-NC, and miR-1204 inhibitor + sh-NR3C2, respectively. **a** NR3C2 expression in U251 cells was detected by RT-qPCR. **b**, **c** The proliferation of U251 cells was examined by CCK-8 and colony formation assays. **d**, **e** The apoptosis of U251 cells was examined by caspase-3 activity and TUNEL assays. GAPDH was used as internal control for NR3C2 expression detection. **P < 0.01, ***P < 0.001

Finally, we probed whether NR3C2 affected the regulation of miR-1204 on GBM cell development. RT-qPCR data presented that miR-1204 inhibitor elevated NR3C2 expression, and then this effect was reversed by sh-NR3C2 in U251 cells (Fig. 4a). The proliferation ability of U251 cells inhibited by miR-1204 inhibitor could be restored by the NR3C2 depletion (Fig. 4b, c). Furthermore, the facilitating effect of miR-1204 inhibitor on caspase-3 activity was counteracted by NR3C2 silencing in U251 cells (Fig. 4d). Likewise, the apoptosis of U251 cells was increased by miR-1240 depletion, and such increase was then abrogated by knockdown of NR3C2 (Fig. 4e). In summary, miR-1204 drove GBM cell proliferation and inhibited cell apoptosis through targeting NR3C2.

## Discussion

Over the past decades, miRNAs have been clarified as effective prognostic and therapeutic targets in cancer by several studies [8–11]. Numerous previous works have unveiled the oncogenic or tumor-repressing behaviors of miRNAs in GBM progression. For instance, miR-1179 suppresses GBM proliferation and cell cycle through targeting E2F5 [20]. MiR-133a promotes TRAIL resistance in glioblastoma through repressing DR5 and NF-κB pathway [21]. MiR-137 suppresses CXCL12 expression, inhibiting glioblastoma progression [22]. Interestingly, miR-1204 have been manifested to drive the progression of breast cancer and ovarian squamous cell carcinoma via regulating cell growth and EMT [24, 39]. Nevertheless, the role of miR-1204 in GBM was poorly comprehended. Accordingly, our study was the first to validate the upregulated miR-1204 level in GBM cell lines. Subsequently, gain-of-function and loss-of-function assays proved that miR-1204 aggravated the proliferation and repressed the apoptosis of GBM cells, indicating the carcinogenic role of miR-1204 in GBM.

As reported by recent works, transcription factors participate in the transcriptional modulation of miRNAs [28, 29]. In this study, we found that CREB1 potentially interacted with miR-1204 promoter through miRGen. Previously, CREB1 has been demonstrated to be involved in the regulation of various malignancies, including GBM [31, 32]. Furthermore, it has been shown that several oncogenic miRNAs are activated by CREB1 transcriptionally, resulting in the facilitation of cancer development [30, 33]. Our study was the first to demonstrate that CREB1 targeted the promoter of miR-1204 to induce the transcription of miR-1204 in GBM.

Existing evidence has revealed that miRNAs target the 3′UTR of mRNAs to trigger translation inhibition and mRNA degradation, so as to result in the post-transcriptional silence of gene expression [15]. In this research, we identified by miRDB that NR3C2 potently interacted with miR-1204. Besides, previous studies have illustrated that downregulation of NR3C2 gene and its protein MR leads to poor prognosis in lung and colon cancer [35, 36]. Also, the tumor-suppressive role of NR3C2 has been validated in pancreatic cancer and clear cell renal cell carcinoma [37, 38]. We were the first to investigate the role of NR3C2 in GBM. The low expression of NR3C2 in GBM samples was obtained through GEPIA. Moreover, our data confirmed that NR3C2 expression was downregulated in GBM cell lines, indicating the potential implication of NR3C2 in GBM. We further revealed that miR-1204 interacted with NR3C2 mRNA and inhibited its expression in GBM. Finally, we confirmed through rescue assays that miR-1204 could promote GBM cell proliferation via inhibiting NR3C2 expression.

## Conclusion

In conclusion, we were the first to reveal that CREB1-induced miR-1204 malignant phenotype of GBM through targeting NR3C2, indicating that miR-1204 may be a promising biological target for GBM treatment.

## Data Availability

Research data will be shared if necessary.

## Abbreviations

GBM: Glioblastoma
ncRNAs: Non-coding RNAs
CREB1cAMP: Responsive element binding protein 1
NR3C2: Nuclear receptor subfamily 3 group C member 2
miRNAs: microRNAs
EMT: Epithelial-to-mesenchymal transition
VDR: Vitamin D receptor
MR: Mineralocorticoid receptor
NHA: Human astrocyte cell
cDNAs: Complementary DNAs
